# Fine-Tuning Shifts Form Before Competence: Treatment-Correlated Style Drift Confounds Surface-Form Evaluation of Adapter Specialization

**DOI:** 10.3390/e28101116

**Published:** 2026-10-13

**Authors:** Othmane Benyoucef, Latifa Dekhici

**Affiliations:** 1 Computer Science Department, University of Sciences and Technology of Oran Mohamed-Boudiaf, Oran 31000, Algeria; 2 LDREI Laboratory, Higher School of Electrical and Energetic Engineering of Oran (ESGEEO), Oran 31000, Algeria

**Keywords:** parameter-efficient fine-tuning, DoRA, DFE-stacking, adapter composition, collaborative decision-making, multi-expert systems, evaluation validity, large language models, Gated DeltaNet, style drift, structured extraction

## Abstract

Stacking task adapters on a frozen domain adapter is a common approach for modular specialization of large language models, usually justified by knowledge injection; whether the surface-form graders standard in this setting can measure such specialization validly has remained unexamined. Two challenges follow: isolating the domain layer’s marginal contribution under identical task training, and determining whether measured differences reflect competence or output form. In this work, both are addressed with a matched-arm DoRA ablation on a frozen Qwen 3.5 9B hybrid: a 242 M-parameter domain expert is trained on a licensed 27.8 M-token corpus, and byte-identical task adapters are trained flat, stacked (DFE-stacking), and jointly across three tasks and five conditions, with the key extraction pair replicated at a second training seed; every grader is audited by per-item failure classification and a dual-rater blind human re-grade with consensus adjudication. The principal finding is that fine-tuning shifts output form before competence: stacking yields no seen-task benefit; an apparent generalization collapse on an unseen schema reduces to a single, highly seed-stable formatting coercion that the domain layer dampens and task-mixture training eliminates, and most automated keyword-grading failures prove to be correct paraphrases, leaving no genuine capability loss detected on any instrument. Because such style drift is induced by the training condition itself, surface-sensitive grader error becomes bias aligned with experimental arms rather than noise. Three additional experiments extend the findings: the coercion replicates on Llama 3.1 8B (60% vs. 19% untuned); a pre-registered 240-item formatting-perturbation battery across four further unseen schemas and eight conventions reveals fine-tuned conditions ranking lowest under strict grading yet highest under format-normalized grading; and a matched-compute wrong-domain control shows the domain layer’s coercion dampening to be generic substrate regularization, while value integrity under the induced drift tracked the substrate’s domain. The principal contribution lies in the identification and quantification of this confound: surface-form evaluation of adapter specialization is structurally confounded, and per-item audits of the type released herein are a necessary component of specialization studies.

## 1. Introduction and Related Works

Large language models are increasingly specialized for domains and tasks through parameter-efficient fine-tuning (PEFT), which trains small adapter modules against a frozen base model. As organizations accumulate specializations, a modular strategy has become attractive: train a high-rank adapter that captures a whole domain, freeze it, and train small task adapters on top of it, swapping task adapters at inference while the domain layer remains always active. The approach’s usual justification is *knowledge injection*: the domain layer stores what the tasks share, so task adapters stacked on it should learn faster or generalize better than task adapters trained on the bare base. This justification carries real costs when acted upon: the domain layer is the most expensive artifact in the stack, and deployment complexity grows with every composed module. Yet the justification is rarely tested in isolation, and, as this paper shows, the instruments used to test it are themselves at risk: fine-tuning changes not only what a model can do but how it writes, and evaluation methods that key on surface form can convert stylistic change into apparent capability change. Whether a measured difference between adapter configurations reflects competence or formatting is, this paper argues, the central validity question for adapter-specialization research.

The broader PEFT literature establishes the components of this approach. Low-rank adaptation (LoRA) [1] and its weight-decomposed refinement DoRA [2] are the standard adapter families; quantized variants [3] make training affordable on commodity hardware, and surveys map the rapidly growing design space [4]. Adapter methods inherit the catastrophic-forgetting problem of sequential training [5], which frozen-substrate designs sidestep by construction. Biderman et al. [6] show that low-rank adapters learn less and forget less than full fine-tuning, motivating frozen-base designs, and Schulman and Thinking Machines Lab [7] show that adequately ranked LoRA matches full fine-tuning under correct hyperparameters. On the architecture side, PEFT behavior on non-standard backbones is only beginning to be mapped: Galim et al. [8] characterize LoRA on state-space models, finding module-dependent effectiveness, while the hybrid Gated-DeltaNet/attention family [9,10,11] used in this study has no published adapter characterization at all. A separate line of work studies evaluation itself: lexical and span-based metrics are known to diverge from human judgments of correctness under paraphrase [12,13,14], model outputs are highly sensitive to surface features of prompts and formats [15], and LLM-based judging carries its own calibration hazards [16,17]. Two further results anticipate the present findings: instruction tuning has been argued to teach primarily style and format rather than knowledge (the superficial-alignment hypothesis) [18], and fine-tuning has been found weaker than retrieval for injecting factual knowledge [19].

Closest to the present subject is the literature on adapter composition. AdapterFusion [20] established non-destructive composition of task adapters on encoder models; MixDA [21] is the direct structural precedent for the present design: domain adapters are trained and frozen, task adapters are trained atop them, and seen-task gains are reported on encoder models. Routed adapter mixtures [22,23] compose many small experts under a learned gate; serving systems [24] address the deployment side. Most recently, Brainstacks [25] composes frozen MoE-LoRA stacks across domains for continual learning and reports that an outcome-trained router dispatches prompts by transferable capability rather than by domain content, evidence, from routing behavior, that what adapters store may not be domain knowledge in the intuitive sense.

These works share three limits that motivate the present design. First, the matched control is missing: studies that stack adapters seldom train the identical task adapter—on identical data and hyperparameters, with and without the domain layer underneath—so the domain layer’s marginal contribution is not isolated; absent that control, practitioners purchase the stack’s most expensive artifact on an untested assumption. Second, behavior beyond the tasks’ training distribution is rarely probed: although generalization is where a shared foundation should matter most, the regime in which the foundation is expected to justify its cost is precisely the one left unmeasured, and deployment-time behavior under distribution shift is first encountered, untested, in production. Third, and most consequentially, the evaluation instruments are not audited: no stacking study identified in this review verifies that its graders can distinguish a model that became less capable from a model that merely writes differently, even though the treatment being studied is precisely one that changes how models write. The evaluation-validity literature, for its part, documents metric brittleness on static benchmarks, where the grader error is roughly constant across the systems compared; it has not examined the acute problem arising when the experimental treatment differentially shifts each arm’s surface distribution, thereby aligning grader error with the experimental conditions. The consequence of this third limitation is field-wide: published comparisons between fine-tuned variants may partly reflect grader bias rather than capability differences in either direction.

This paper addresses all three limits in one design. The matched-arm ablation is run on a frozen Qwen 3.5 9B hybrid: a 242 M-parameter DoRA domain expert trained on a licensed, decontaminated data-analytics corpus, then byte-identical task adapters trained flat versus stacked (the DFE-stacking configuration), alongside domain-only, base, and jointly trained multi-task arms: five conditions, three tasks, and a second seed replication of the key extraction pair. Behavior beyond the training distribution is probed with pre-planned splits: an unseen renderer, an entirely unseen extraction schema, and unseen table types. Every grader is audited with per-item failure classification and a dual-rater blind human re-grade with consensus adjudication. The contributions are: (1) a matched-arm ablation of domain-then-task adapter stacking that isolates the domain layer’s marginal effect on seen tasks and out of distribution; (2) evidence, across three independent grader families with distinct failure mechanisms (paraphrase sensitivity, register, format fidelity), that treatment-correlated style drift masquerades as capability change in both directions, replicated on a second base model and quantified per formatting convention by a pre-registered perturbation battery, alongside a released blind-review protocol and per-item failure decompositions that make such audits cheap to replicate; (3) an honest cost accounting of modular versus joint training at small task counts; and (4), to our knowledge, the first systematic operational characterization of DoRA on a Gated-DeltaNet hybrid, including an upstream stop-token misconfiguration affecting default generation with the released checkpoint configuration. Task datasets, adapter weights for every condition and seed, all evaluation and figure-generation code, and the re-grade protocol and consensus adjudication records are released. Beyond its immediate findings, this study connects to the broader problem of collaborative decision-making in multi-expert systems: the architecture under test is a society of specialized experts whose composition must be decided at inference time, and the dual-rater consensus protocol developed for evaluation is itself a small collaborative decision procedure with an explicit disagreement-resolution rule.

The remainder of this paper is organized as follows. Section 2 formalizes the research questions and experimental conditions. Section 3 presents the architecture, training protocol, datasets, and evaluation instruments. Section 4 reports the results and discusses the five findings together with the operational characterization. Section 5 concludes with limitations and perspectives. Appendix A, Appendix B, Appendix C and Appendix D document the instrument validation history, reproducibility materials, the blind re-grade protocol, and the extended operational record.

## 2. Problem Statement

Let $f_{\theta }$ be a frozen base language model, *D* a high-rank domain adapter trained once on a domain corpus and thereafter frozen, and $T_{1},\ldots ,T_{N}$ small task adapters. The approach under test deploys $f_{\theta }+D+T_{i}$, with $T_{i}$ swapped per request; each $T_{i}$ is trained with *D* merged into its substrate. The knowledge-injection justification predicts that a task adapter trained on $f_{\theta }+D$ (*stacked*) should match or exceed the same adapter trained on $f_{\theta }$ alone (*flat*), with the largest advantages where task-specific training data is least sufficient, in particular, under distribution shift.

The test is formalized as three research questions.

**RQ1 (seen-task effect).** With task data, hyperparameters, initialization, and training seed held byte-identical between arms, does the stacked configuration outperform flat on the tasks’ held-out test sets? The knowledge-injection account predicts yes; a null here means rank-adequate task adapters extract what they need from task data alone, and the domain layer’s cost buys no trained task quality.

**RQ2 (out-of-distribution effect).** Do the arms differ on generalization splits designed before training—a third, unseen prose renderer for extraction; an entirely unseen extraction schema whose keys never occur in training; or unseen table types and phrasings for summarization? If differences appear, the question becomes mechanistic: are they differences in capability, or differences in something else that the grader registers as capability?

**RQ3 (measurement validity).** Can the surface-form graders standard in this setting (sandboxed execution for code, exact field match for extraction, verbatim containment for summarization, lexical overlap for open QA) measure RQ1 and RQ2 without confounding, given that fine-tuning may shift register, formatting conventions, and answer style along with, or instead of, competence? RQ3 is usually treated as a methodological footnote; the present design treats it as a first-class question, with every instrument audited by per-item failure classification and a dual-rater blind human re-grade.

Five conditions per task instrument these questions: *base* (frozen model only), *domain* (domain expert only), *flat*, *stacked*, and *joint* (one adapter trained on the concatenation of all three tasks’ data). The base and domain arms anchor what the substrate contributes without task training; the flat/stacked pair isolates the domain layer’s marginal effect under identical task training; the joint arm prices the modular design against the simplest alternative (one adapter, all data) on both quality and cost. The extraction-task flat/stacked pair is additionally replicated end-to-end at a second training seed so that any out-of-distribution difference can be checked for seed stability before it is interpreted.

The scope boundaries are stated in advance. The study covers one base model (a hybrid Gated-DeltaNet/attention decoder, itself uncharacterized for DoRA and therefore requiring the operational validation reported alongside), one domain (data analytics), three tasks, and N=3 for the modular-versus-joint comparison; it does not test routed multi-expert serving, the task count at which joint training begins to interfere, or cross-domain composition. Within that scope, the pre-registered expectation was a confirmation of RQ1’s prediction with possible RQ2 effects. The experiments instead return a null on RQ1, an RQ2 difference whose mechanism proves to be entirely surface-formal, and an answer to RQ3 that reframes the study: the graders’ errors are not noise but bias aligned with the experimental arms, because the treatment itself moves each arm’s surface distribution. Establishing that reframing, together with what it implies for how adapter specialization should be evaluated, is the substantive problem this paper addresses.

## 3. Methods

### 3.1. Architecture

DFE-stacking (Domain-Foundation-Expert stacking) composes a frozen base (Figure 1);

a **Domain Foundation Expert** (DoRA adapter of rank r=96 and scaling factor α=96, where *r* is the dimensionality of the low-rank update and α scales its contribution; 242.07 M parameters, 2.63% of base), trained once on the domain corpus, then frozen;
and per-task **Task Experts** (QDoRA, i.e., DoRA trained over a 4-bit-quantized base; r=48, α=48; 121.7 M parameters), trained with the domain expert merged into the substrate and only the task adapter trainable (enforced by runtime asserts over the freeze map). Adapters target 10 module classes across both attention types: DeltaNet projections (in_proj_qkvz components, out_proj), full-attention q,k,v,o_proj, and MLP gate,up,down_proj. The low-dimensional DeltaNet gates in_proj_a/b (4096→32) are excluded, since useful ranks would exceed the original weight rank.

### 3.2. Training Protocol

Domain: BF16 (bfloat16) precision, one epoch, learning rate $10^{-4}$ with cosine decay and 3% linear warmup (warmup_ratio=0.03; 50 optimizer steps for the domain run), effective batch 32, loss masked to assistant tokens, over-cap sequences dropped rather than truncated (end-of-turn supervision never censored); 26.9 h on a single NVIDIA L40 GPU (46 GB usable); task experts fit 24 GB-class GPUs.

Task: NF4 (4-bit NormalFloat quantization) QDoRA, two epochs, learning rate $10^{-4}$, effective batch 16; flat and stacked use byte-identical data, hyperparameters, and seed, differing only in substrate.

Joint: identical configuration on the shuffled three-task concatenation (8936 records). All evaluation uses merged BF16 weights and one decoding protocol (Section 4.8).

### 3.3. Data

#### 3.3.1. Domain Corpus

Totals 27.78 M training tokens: 78% Stack Exchange (CC BY-SA; Cross Validated quality-rank-capped, Data Science, AI, keyword-quota-filtered Stack Overflow), 22% official documentation of 22 open-source projects (cloned August 2026), balanced over a 10-area taxonomy. Template-only extraction (no LLM anywhere in the corpus), junk filtering, exact + MinHash near-deduplication, and n-gram decontamination against all seven evaluation sources with adaptive shingle widths (5–13 words; fixed 13-grams are blind to short questions); audit log: 7 removals, hand-verified. Per-source license and token accounting ships with the release.

#### 3.3.2. Task Datasets

Each task provides 3000/300/300 train/validation/test records; generalization splits fixed before training. **T1, domain code generation** (organic): Stack Overflow Q&A, question-ID-disjoint from the corpus, quality filtered, decontaminated against HumanEval/MBPP at mining time. A v1 mining filter admitted ∼22% non-Python items (detected during evaluation; disclosed); released **v2** (mandatory Python-ecosystem conjunct, other-language negative filter) reduces detectable leakage to 0/300 test items. Reported T1 numbers use v2; v1 replicates the qualitative pattern (Appendix A). **T2, structured extraction** (synthetic, exact ground truth): structured seeds rendered to prose by templates, so field-level truth is exact by construction. Training: four schemas, two renderers. Test: a third unseen renderer (200 seen-schema items) plus a fifth, fully unseen schema, model_deployment (100 items) whose keys never occur in training, and whose version field carries a v prefix, a convention absent from training targets, which Section 4.7 shows matters more than the schema itself. **T3, analytical summarization** (synthetic, verifiable): markdown tables with derived-fact targets stored per item for verbatim-verifiable grading; test includes unseen phrasings and an unseen table type. An illustrative test item, quoted from the release: the input is a model-comparison table (one row per model: name, accuracy, training minutes; here mlp 0.836/205, lightgbm 0.626/102, xgboost 0.714/40); the stored targets are derived facts such as best_model = mlp, best_accuracy = 0.836, runner_up = xgboost, margin = 0.122, and fastest_model = xgboost; the item’s reference summary reads “Top of the table: mlp, at 0.836 accuracy, 0.122 clear of xgboost. Bottom: lightgbm (0.626). Quickest training run: xgboost.”; a model’s summary is graded by verbatim containment of each stored fact, so every credit is mechanically verifiable against the table.

### 3.4. Experimental Setup

The experimental grid is instrumented as follows.

**Conditions.** base/domain/flat/stacked/joint; 15 grid cells at seed 42; the T2 flat/stacked pair replicated end-to-end at training seed 43.

**Graders.** T1: fenced code executed in a sandbox pre-loaded with the scientific Python stack; pass = error-free execution; symmetric static exclusions (non-Python, SQL-only, unavailable heavy frameworks, cap-truncated mid-code block), every count reported per cell. T2: strict JSON parse, exact key set, exact per-field values; we additionally report a *format-normalized* variant, defined post hoc after per-item failure classification (Section 4.7). These operationalize two distinct constructs, both valid and both reported: strict exact match measures *schema/value fidelity* (the production-relevant outcome), while the normalized variant measures *semantic extraction correctness*; the two are not interchangeable, and each result is interpreted against the construct its grader measures. T3: verbatim containment of stored facts. On representativeness: exact-match field grading and execution-based grading are the field-standard families for extraction and code respectively; the keyword tier is a pragmatic instrument whose failure mode replicates the documented behavior of the standard lexical-overlap family (EM/F1) [12,14]. The confound mechanism analyzed in Section 4.7 is grader-agnostic and applies to the standard instruments, not only ours.

**Open-ended domain evaluation.** A 500-item open-ended question set in the corpus domain (knowledge and code-generation questions), answered by both the base model and the domain expert under the identical decoding protocol, graded automatically by a keyword-containment tier and audited by the blind dual-rater human protocol of Appendix C.

**External instruments.** HumanEval [26], MBPP [27] (lm-eval, greedy, batch 8) per condition; full-suite MMLU [28] and ARC-Challenge for retention (base anchor: MMLU 78.65, HE 69.5, MBPP 55.4).

**Statistics.** Wilson 95% intervals on proportions; exact McNemar on paired discordants; greedy decoding throughout; “seed” refers to training stochasticity.

## 4. Results and Discussion

### 4.1. Retention

Injecting 242 M trained parameters across every layer produced no measurable retention cost: full-suite MMLU 78.61 vs. base 78.65 (Δ=−0.04); machine-learning subscore +0.89; ARC-Challenge acc_norm +1.28.

### 4.2. Main Grid

Table 1 reports the full experimental grid; the rows most consequential for the analysis are the two unseen-schema readings and the coercion rate.

The strict unseen-schema row shows disjoint Wilson intervals for flat 27 [19.3, 36.4] vs. stacked 62 [52.2, 70.9]; the normalized row shows the difference is almost entirely one formatting convention. Per-item classification of every unseen-schema failure in every condition and both seeds (242 failures total) finds 237 are the identical coercion—with the v prefix stripped from the version string—occasionally coerced further to a bare JSON number; the remaining five are isolated single-field errors (two in domain, one each in flat seed-42, flat seed-43, and joint). Separately, the *seen*-schema misses of base and domain are not formatting: they concentrate on inferential fields (e.g., winner: null where the target convention is "inconclusive" for non-significant tests; missed target_column), indicating that task training’s seen-task value here is *convention acquisition for inferential fields*, not raw extraction ability.

### 4.3. Seed Replication

Retraining the T2 pair at training seed 43: flat again passes exactly 27/100 strict, item-identical to seed 42, with 297/300 identical greedy outputs from SHA-distinct adapter weights, and 72/73 failures again the version coercion, while stacked passes 55/100 strict with all 45 failures version-only (coercion rate 45% vs. 38% at seed 42). The coercion is a highly seed-stable property of the flat training configuration; the domain layer’s dampening replicates (+28–35 strict points; coercion 72% → 38–45% across seeds).

### 4.4. Generality and Mechanism: Cross-Model, Cross-Convention, and Cross-Substrate Probes

Three further experiments probe the generalizability of the extraction findings beyond a single base model, a single formatting convention, and a single substrate domain; decision criteria for all three were specified prior to execution.

**Cross-model probe.** The flat T2 configuration was retrained on Llama 3.1 8B Instruct, a standard full-attention transformer from a different family using a different tokenizer: byte-identical task data and seed, the same QDoRA recipe (r=48, α=48, NF4, two epochs, learning rate $10^{-4}$; 127.2 M trainable parameters across the seven standard projection modules, close to the hybrid’s 121.7 M), and evaluation by the same grader under Llama’s native chat template and stop tokens. On the unseen schema, the strict score falls from the untuned model’s 77/100 to 36/100, and the version-coercion rate rises from 19% to 60% (two-proportion z=5.9, $p<10^{-8}$), while three of the four trained schemas saturate (136/136). Against the pre-registered criteria (reproduces: fine-tuned coercion ≥30% *and* ≥5× the same model’s base; does not reproduce: ≤base +5 points), the outcome is formally intermediate, and we report both interpretations: the absolute arm is cleared decisively, while the ratio arm is not, because untuned Llama already coerces at 19% where untuned Qwen coerced at 8%. The expression is model-specific: Llama predominantly coerces the string to a JSON *number* ("v4.19"→ 4.19) where Qwen strips to a bare string. Two additional observations complete the record. Both untuned bases share a label-boundary confusion on the one inference-requiring field (writing null where the target convention is "inconclusive"; 13/64 items each), providing evidence that the audited behaviors are not idiosyncrasies of one model family. And fine-tuning on Llama, unlike on Qwen where every trained condition solved that field perfectly, introduces a genuine decision regression there (35 of its 36 errors declare no winner where one exists); this is a competence-related training cost, and we report it as distinct from the form confound rather than subsumed by it.

**Formatting-perturbation battery.** A 240-item battery, generated and annotated prior to the evaluation of any condition, comprises four fully unseen schemas (60 items each, two prose renderers) whose fields carry eight formatting conventions (v-prefixed semver, ISO date, leading-zero identifier, unit-suffixed duration, unit-suffixed memory, uppercase severity label, Boolean literal, quoted numeric port). Every convention-sensitive value appears verbatim in its passage, and each item stores the canonical value together with its anticipated coerced forms, so coercion is classified mechanically rather than by inspection. Table 2 reports strict and format-normalized scores with coercion rates for the three conventions that saturate.

**Wrong-domain control.** To disentangle domain-specific content effects from generic frozen-substrate regularization, a control substrate of identical rank, precision, schedule, and training compute (r=96, BF16, one epoch, matched token count) was trained on an unrelated-domain corpus (system administration and networking: Server Fault, Super User, and Unix & Linux Stack Exchange plus ten infrastructure projects’ documentation; 26.9 M tokens) built by the same pipeline under the same seven-source decontamination, and the byte-identical T2 task adapter was retrained stacked on it at the same seed. The pre-registered comparator was the in-domain stack’s coercion rate (38–45% across seeds; decision band ±10 points). On the unseen schema, the wrong-domain stack passes 55/100 strict with a version-coercion rate of 43% inside the band: the dampening that F2 attributes to the domain layer is reproduced by an unrelated substrate of matched compute, so the coercion dampening is *generic* frozen-substrate regularization rather than a domain-content effect, and F2’s interpretation states the claim in this form. The control substrate alone behaves like the untuned base (93/100 unseen strict; 5% coercion; the same inferential-field misses on seen schemas), supporting the control’s validity. The battery, however, reveals a dimension on which the substrates are not interchangeable (Table 2): the wrong-domain stack’s normalized score is 79.2 versus the in-domain stack’s 98.8, with the residue concentrated in form-driven *value corruption* (durations truncated in attempted conversion, "40m23s"→40; semver squashed to pseudo-decimals, "v1.7.8"→1.78) plus the only parse failures of any Qwen condition (6/240). Under distribution shift, the in-domain stack coerced form while preserving every underlying value; the wrong-domain stack coerces form *and* corrupts values. We therefore split the original claim: the *rate* of format coercion is stabilized by any matched-compute frozen substrate, while *content integrity under the induced form drift* measurably tracked the substrate’s domain in our setting. Three results follow from Table 2. *First, strict grading inverts the ranking.* The strict ordering (base 75.0 > joint 72.5 > domain 67.5 > stacked 48.8 > flat 42.1) suggests specialization damaging the specialists’ own task family, yet every fine-tuned Qwen condition scores *above* base once form is normalized (97.5–100.0 vs. 94.2). The specialists extract the underlying values better than base, and the entire 33-point strict spread is output form. *Second, the coercion fingerprint is near-deterministic and cross-model.* Flat, stacked, and Llama-flat coerce the same three conventions at exactly 100% (60/60 each) while leaving dates, identifiers, severity labels, and Booleans essentially untouched: task fine-tuning does not loosen formatting so much as install absolute rules, and it installs the same three on both architectures. *Third, the mitigation replicates.* The joint adapter again sits closest to base strictly, reaches 100.0 normalized, and coerces the semver convention below base’s own rate (36.7% vs. 45.0%), extending the main grid’s joint-suppresses-coercion result to schemas nothing was trained on. Two further observations clarify the mechanism. The domain-only condition, which saw no task data at all, coerces unit-suffixed memory at 73.3% versus base’s 31.7%: domain-only training alone shifts output form, the paper’s title claim isolated from task supervision. And Llama-flat frequently corrupts the value in the act of reformatting it ("40m23s"→ 40323, an incorrect unit conversion of a string present verbatim in the passage): form drift introduces content errors, and because such items evade the coercion classifier, the form-attributable shares reported here are conservative lower bounds.

### 4.5. Blind Semantic Re-Grade

Table 3 summarizes the dual-rater blind re-grade of the open-ended domain evaluation.

The expert’s knowledge answers are 96.1% lenient-correct pooled across both raters (no factual damage); its residual deficit concentrates in code answers graded as standalone artifacts (72.9% vs. 85.0%), which is the register signature of Section 4.7.

### 4.6. Cost Accounting

Table 4 prices the three training strategies.

At the evaluated task count (N=3, one domain), joint training was simultaneously strongest on quality and cost; stacking is not a training economy at this scale. Its measured returns are surface fidelity stabilization and O(1) expert addition.

### 4.7. Findings

Five findings organize the analysis; each is stated with its evidence and the audited failure modes behind it.

#### 4.7.1. F1: Stacking Does Not Improve Seen-Task Quality

Both arms saturate T2’s seen schemas under an unseen renderer (200/200); both score 45.7% on T3 all-facts with per-type profiles matching within one item; T1 paired discordance is 5-vs-3 (McNemar p=0.73), structurally replicated on the independently mined v1 test set. Rank-48 adapters learn these behaviors from 3000 examples without help. Practitioners paying domain-adapter costs on the expectation of seen-task gains should expect none at this granularity.

#### 4.7.2. F2: The Unseen-Schema Decomposition and What the Domain Layer Actually Stabilizes

Read naively, the strict unseen-schema column is a catastrophic generalization result: task training drops the base’s 92/100 to 27/100, and the frozen domain layer rescues half the loss. Per-item classification dissolves and then reconstitutes the finding at the correct size. Every flat failure but one is the same event: the source passage says "version": v7.12; the model writes 7.12. No trained schema contains a version field; T2 training targets are dominated by bare numerics (rates, counts, latencies); the pattern is consistent with the adapter acquiring a *value-normalization prior* that is applied to the first version-like string encountered out of distribution; the wrong-domain control reported in Section 4.4 confirms the substrate side of the mechanism causally, and dose response remains scoped in Section 5. The worked example (item 4; passage contains v7.12 verbatim) is:
**flat**:{"model": "resnet50-ft", "version": "7.12", …}**stacked**:{"model": "resnet50-ft", "version": "v7.12", …}

Three measured properties give the finding its shape (Figure 2). *Seed stability:* independently seeded flat runs converge to item-identical behavior (297/300 identical greedy outputs; the same 27/100 strict passes) from SHA-distinct weights; the coercion behaves as a property of the objective and data rather than a draw. *Dampening:* the frozen domain substrate roughly halves the coercion rate (72% → 38–45% across seeds), with stacked’s failures being a strict subset of flat’s at seed 42. *Elimination by diversity:* the joint adapter, trained on the *identical* T2 records, mixed with code and summarization data, coerces 0%. Cross-task diversity, not schema variety, is the strongest protection. Under format-normalized grading all conditions score 98–100: we find no evidence that any condition lost the *ability* to extract the unseen schema. We therefore claim surface fidelity stabilization, not generalization rescue, as DFE-stacking’s measured out-of-distribution effect: the frozen substrate regularizes *output form* under distribution shift—a dampening the wrong-domain control (Section 4.4) shows to be substrate-generic rather than domain-specific—while content integrity under the induced drift tracks the substrate’s domain. The claim is smaller than the stacking literature’s and arguably better evidenced, as every causal link (training-target statistics → learned prior → out-of-distribution application → substrate dampening) is measured. It is also operationally real: 7.12 for v7.12 is a genuine fidelity failure in production extraction pipelines, and a 72%-vs-0% gap between architectures is a deployment-relevant difference. Convention breadth and cross-model generality of the coercion are established in Section 4.4. Mechanism questions we scope for follow-up: dose-response of the coercion with training epochs, as well as whether richer format diversity within a single task’s training targets substitutes for cross-task diversity.

#### 4.7.3. F3: Three Graders, One Confound

The extraction decomposition is the third instance of the study’s through-line, and the three together instantiate the title’s claim: the treatment moved each arm’s output form, so each grader’s form-sensitivity converted style distance into apparent capability difference, bias, not noise. *Keyword grading* (Table 3): ≈87–88% of the expert’s automated failures are correct paraphrases (consensus-adjudicated, dual-rater); passes are only ∼63–65% strictly correct; the instrument errs in both directions and the true gap is 2–3 points. A representative item from the blind-reviewed queue is as follows:

**Q:** What is KL divergence?

**Required keywords:** distance, distributions

**Expert answer (keyword-graded FAIL; blind human verdict:** ***correct*****, high confidence):** “The KL divergence is a measure of the *difference* between two probability distributions. It is defined as $D_{KL}\left(P∥Q\right)=\sum_{i}P\left(i\right)\log\frac{P(i)}{Q(i)}$ … non-negative and zero iff *P* and *Q* are identical … asymmetric, $D_{KL}\left(P∥Q\right)\neq D_{KL}\left(Q∥P\right)$.”

The answer is correct and complete; it fails on the synonym *difference*/*distance*. (A second mode: a fully correct tensor.to(’cuda’) snippet fails the keyword device.) *Standalone execution*: the condition ordering tracks register-signal concentration monotonically (base 30.0> joint 23.5> domain 21.3> flat 19.4≈ stacked 18.9); the domain-only condition answers 65/300 code questions in unfenced prose (base: 12), which is the forum explainer register in its purest observable form; yet on natively self-contained benchmarks, the deficit vanishes or inverts (flat MBPP +5.4 over base). *Exact-match extraction*: a single learned formatting convention manufactures a 65-point “collapse.” The general warning is: any fine-tune that shifts register or format will look damaged (or occasionally improved) under surface-form grading calibrated to another style, the artifact can be as large as the effect being measured, and only per-item inspection distinguishes the two. We release the blind-review protocol and per-item classifications to make that inspection cheap for others.

#### 4.7.4. F4: The Joint Baseline, Reported Straight

Joint wins or ties everything at N=3: seen-task ceilings, ≈0% coercion (better surface fidelity than base), best trained-condition HumanEval (67.1), mildest execution-register cost (23.5), total cost equal to the flat sum with no domain prerequisite (Table 4). Three same-domain tasks at 9000 mixed examples produce no measurable interference; data diversity delivers architecturally what stacking delivers structurally. The case for modularity at this scale is operational: per-expert governance, rollback, O(1) addition, plus F2’s stabilization. The task count at which joint interference emerges is the decisive open experiment.

#### 4.7.5. F5: Composition Cost Is Inherited, Not Created

Stacked trails flat on external benchmarks (HE −5.4, MBPP −3.0), which reads as a stacking tax until the domain-only row decomposes it: the domain layer itself trades HumanEval-style completion for MBPP-style spec-following (HE 69.5→61.6; MBPP 55.4→61.4, the best MBPP of any condition), and stacked inherits the profile nearly unchanged (within 2.4 points of its substrate on HE). Composition adds ≤2–4 points; the profile shift originates in domain training, is plausibly register-mediated, and is a property chosen with the corpus, not a hidden mechanism cost.

### 4.8. Operational Findings on the Gated-DeltaNet Hybrid

Three findings any practitioner fine-tuning this architecture will encounter (full operational record in Appendix D) are described as follows.

**Stop-token misconfiguration (upstream; released checkpoint configuration).** The released Qwen3.5-9B repository ships no generation_config.json, and its config.json declares a scalar eos_token_id (248044) omitting the chat turn terminator <|im_end|> (248046). Default model.generate therefore never stops at end of turn: generation continues into self-generated follow-on turns, repetition, or drift, which downstream resembles capability failure, and which length caps convert into silent truncation. The flaw propagates: merge_and_unload + save_pretrained writes the incomplete configuration into merged artifacts. Mitigation: pass the full stop list at every generate call site; author a correct generation_config.json into every saved artifact.

**Unmerged-generation cost.** Generating through a live DoRA adapter costs ≈10× wall-clock versus merged weights (183→16 s/item): the magnitude renormalization executes per forward pass. Merged evaluation is a requirement at scale; true hot-swap DoRA serving must pre-merge per expert or amortize the renormalization, a concrete systems problem this architecture inherits.

**Merge fidelity.** In total, 8/10 long greedy continuations match exactly between merged and unmerged; the two divergences begin deep into generation, consistent with BF16 associativity drift.

## 5. Conclusions and Perspectives

This work examined the matched control that the domain-then-task adapter stacking approach usually skips, probed behavior beyond the training distribution, and audited every evaluation instrument at the item level. Five findings resulted. First, stacking bought no seen-task quality: byte-identical task adapters reached the same performance with and without the frozen domain substrate. Second, the dramatic out-of-distribution gap between configurations was real, replicated across seeds, and highly seed-stable, yet it decomposed into a single learned formatting convention, which the frozen domain layer dampened, task mixture diversity eliminated, and which exact-match grading, if read as a measure of semantic ability rather than schema fidelity, would misreport as generalization collapse. Third, across keyword, execution, and exact-match instruments, style and format transfer was found on every instrument, and no evidence of genuine capability loss was found under the evaluated instruments after accounting for the identified formatting and register effects; because the treatment itself shifted each arm’s surface distribution, grader error was aligned with the experimental conditions rather than random. Fourth, a jointly trained adapter matched or exceeded every modular configuration at equal training cost, locating the measured value of the modular design in surface fidelity stabilization and constant-cost expert addition rather than in quality. Fifth, the composition cost visible on external code benchmarks was inherited from the domain layer’s own profile shift rather than created by stacking. Three further experiments extend and sharpen the central result: extraction coercion reproduces on a second architecture (Llama 3.1 8B, with a model-specific numeric expression and a decision-field regression that Qwen does not share); a pre-registered 240-item formatting-perturbation battery across four additional unseen schemas and eight conventions demonstrates the strict-versus-normalized inversion at breadth, with the same three conventions coerced at exactly 100% by every task-fine-tuned condition on both models; and a matched-compute wrong-domain control indicates that coercion dampening is generic frozen-substrate regularization (43% versus the in-domain 38–45%), while content integrity under the induced form drift tracked the substrate’s domain. The constructive implication for practitioners follows directly: adapter specialization should be measured with instruments that cannot mistake form for substance; the largest visible effect of fine-tuning should be expected to be stylistic; and a DFE-stacking domain foundation should be paid for only for what it measurably provides, while joint training was the strongest configuration on quality and cost at the evaluated task count.

### 5.1. Limitations

Several limitations bound the claims.

**Synthetic structure in T2/T3.** The coercion finding’s arena is template generated; template regularity plausibly amplifies both the determinism and the uniformity of the failure mode. A naturalistic extraction evaluation (README/model-card passages, verified targets) remains the most critical pending robustness check and is outlined in Perspectives; the perturbation battery (Section 4.4) widens schema and convention coverage but retains its template-generated structure.

**Convention breadth.** Breadth across conventions and schemas is established by the perturbation battery (Section 4.4: four further unseen schemas, eight conventions), though the battery shares T2’s template-generated structure and therefore inherits the synthetic-structure limitation above.

**Scale.** Three tasks in one domain cannot decide modular-vs-joint; the interference crossover experiment is scoped but unexecuted.

**Re-grade agreement.** Dual-rater validation shows high raw agreement (91.8% binary) with moderate unadjusted κ (0.612), driven by the prevalence paradox (PABAK 0.836, AC1 0.896); disagreements concentrate on the partial credit boundary, and the 22 metric-relevant ones were consensus adjudicated with zero tie-breaks. Residual rater subjectivity on partial credit remains, though it does not affect the lenient-binary metrics. More fundamentally, competence is operationalized here through instruments and human judgment that are themselves imperfect: the human anchor is validated by dual rating and consensus adjudication but remains a lenient, coarse measure, and the constructs of semantic correctness, task success, and surface conformity are distinguished by definition rather than by an infallible oracle.

**Mechanism.** The wrong-domain control (Section 4.4) resolves the substrate side of the mechanism; the dose-response of the coercion with training epochs remains future work, as does replication of the content-integrity difference between substrates beyond one task and seed.

### 5.2. Perspectives

The extended version of this work will pursue the deferred experiments: the task-count crossover at which joint training begins to interfere (the decisive experiment for modular-versus-joint at scale); the dose-response of the formatting coercion with training epochs; a naturalistic extraction evaluation on organic text; and format-diversity augmentation as a cost-efficient substitute for cross-task diversity. Router-based orchestration of multiple task experts, the original engineering motivation for the modular design, remains future work and is best understood as a problem of collaborative decision making among specialized agents: a router over experts is a collective decision mechanism whose vote allocation must rest on what each expert measurably contributes, whereas the present results show that such decisions would otherwise rely on capabilities that are substantially stylistic. The dual-rater consensus protocol developed herein, with its pre-committed rubric and conservative tie-break rule, likewise instantiates collaborative judgment under disagreement and offers a reusable template for it. Evaluation of any such orchestration must employ the form-insensitive instruments this paper develops.

## Figures and Tables

**Figure 1:**
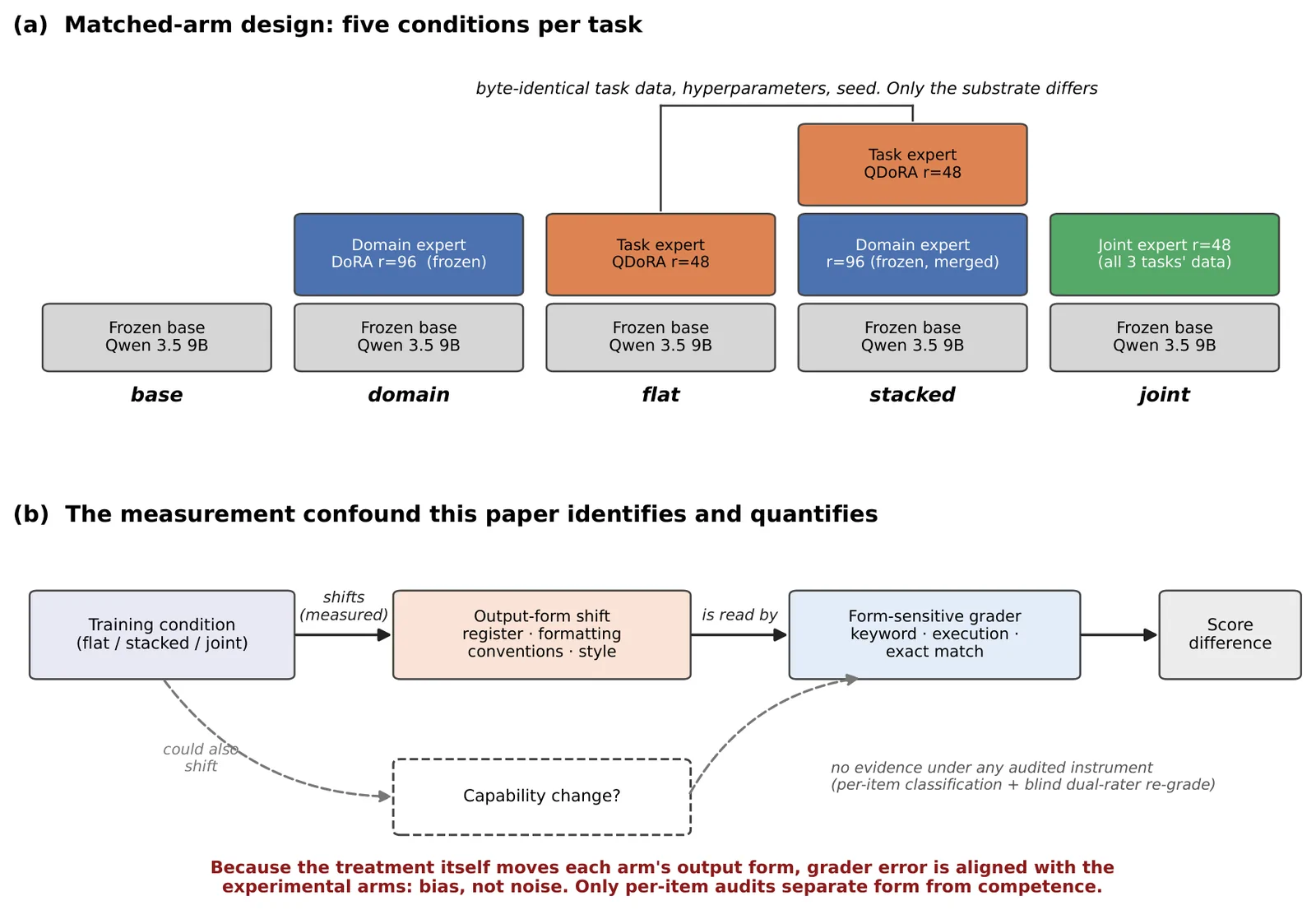
(**a**) The matched-arm experimental design: five conditions per task, with the flat and stacked arms trained on byte-identical task data, hyperparameters, and seed so that the frozen domain layer’s marginal effect is isolated. (**b**) The measurement confound this paper identifies and quantifies: the training condition itself shifts output form; form-sensitive graders read that shift as a score difference; grader error is therefore aligned with the experimental arms. The capability pathway (dashed) shows no evidence under any audited instrument.

**Figure 2:**
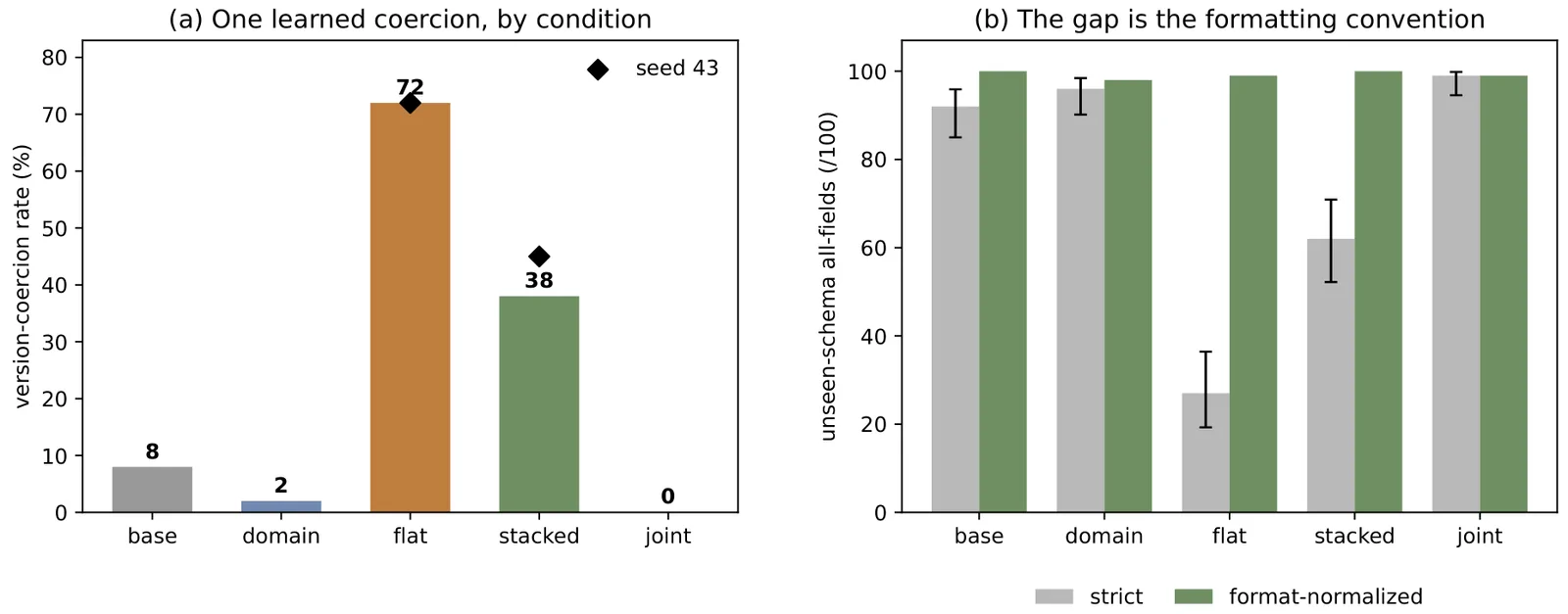
One formatting convention accounts for the entire unseen-schema gap. (**a**) Version-coercion rate by condition (diamonds: seed-43 replication). (**b**) Strict vs. format-normalized unseen-schema scores; error bars are Wilson 95% intervals on strict.

**Table 1:** Main results (test N=300/task; T2 split into 200 seen-schema items under an unseen renderer and 100 unseen-schema items). “T2 unseen, normalized” re-grades the unseen schema with version-format normalization (Section 4.7). Bold marks the two rows on which the paper’s analysis turns.

Metric	Base	Domain	Flat	Stacked	Joint
T1v2 exec pass (%)	30.0	21.3	19.4	18.9	23.5
T2 all-fields, seen schemas (/200)	177	182	200	200	200
T2 all-fields, unseen schema, strict (/100)	92	96	27	62	99
T2 unseen, **format-normalized** (/100)	**100**	**98**	**99**	**100**	**99**
T2 **version-coercion rate** (%)	**8**	**2**	**72**	**38**	**0**
T3 all-facts (%)	0.7	3.7	45.7	45.7	45.7
T3 mean fact recall (%)	31.3	49.1	67.9	66.6	67.9
HumanEval pass@1 (%)	69.5	61.6	64.6	59.2	67.1
MBPP pass@1 (%)	55.4	61.4	60.8	57.8	60.0

**Table 2:** Formatting-perturbation battery (N=240; four unseen schemas × 60 items; generated and annotated before evaluation). Strict denotes an exact key set and exact field values; normalized grants form-forgiving credit on the annotated conventions while remaining exact elsewhere. Convention columns present coercion rates (%, events over 60 opportunities) for v-prefixed semver, unit-suffixed memory, and quoted numeric port. The remaining five conventions are coerced at 0% by every Qwen condition except unit-duration (flat 21.7%; wrong-domain stacked 20.4%; stacked 0%), and at ≤8.5% by Llama-flat. The wrong-domain row additionally has 6/240 parse failures, the only ones of any Qwen condition. Llama rows use that model’s native template and stop tokens.

Condition	Strict (%)	Normalized (%)	Semver	Memory	Port
base	75.0	94.2	45.0	31.7	1.7
domain	67.5	98.3	50.0	73.3	5.0
flat	42.1	97.5	100.0	100.0	100.0
stacked	48.8	98.8	100.0	100.0	100.0
joint	72.5	100.0	36.7	73.3	18.3
wrong-domain stacked	28.3	79.2	85.0	100.0	100.0
Llama 3.1 8B base	51.7	82.9	63.3	83.3	83.3
Llama 3.1 8B flat	23.3	77.5	100.0	100.0	100.0

**Table 3:** Blind re-grade of the 500-item open-ended domain evaluation. Two independent blind raters (268 items dual-rated; 536 reviews), with the 22 metric-relevant disagreements resolved by consensus adjudication (zero tie-breaks); all figures are item-level under consensus > rater-1 precedence. Review coverage of keyword-failures is near-census (156/161 expert, 52/57 base); corrected estimates count unreviewed items as failures. Artifact rate = automated keyword failures rescued as correct/partially correct.

	Base	Domain Expert
Automated keyword score	88.6%	67.8%
Artifact rate (all reviewed kw-fails)	88.5% [77.0–94.6]	87.2% [81.0–91.5]
Corrected, one-sided (N=500)	97.8% [96.1–98.8]	95.0% [92.7–96.6]
Corrected, two-sided lenient	≈94.8%	≈92.7%
Calibration: both-pass strictly correct	65.0% (consensus)	
Calibration: both-fail strictly correct	58.3% (consensus)	
Genuine differences among discordants	20 / 148	

**Table 4:** Training and deployment costs. “Deployed adapter params” counts adapter parameters only, per deployed task configuration; the frozen 9B base model is shared by all conditions and excluded.

	Flat	Stacked	Joint
Upfront (domain expert)	–	26.9 h	–
Per-task training	2.4–3.3 h	2.4–3.3 h	–
Three-task total	≈7.5 h	≈34.6 h	7.7 h
Deployed adapter params	121.7 M	363.8 M	121.7 M
Marginal cost, task N+1	1 small training	1 small training	full retrain + re-validation

## Data Availability

Code and evaluation artifacts: https://github.com/obenyoucef/fine-tuning-style-drift (accessed on 7 October 2026); Adapter weights on Hugging Face: OthmaneBen/dfe-domain-expert-r96, OthmaneBen/dfe-task-t1code-v2-flat-s42, OthmaneBen/dfe-task-t1code-v2-stacked-s42, OthmaneBen/dfe-task-t1code-v1-flat-s42, OthmaneBen/dfe-task-t1code-v1-stacked-s42, OthmaneBen/dfe-task-t2extract-flat-s42, OthmaneBen/dfe-task-t2extract-stacked-s42, OthmaneBen/ dfe-task-t2extract-flat-s43, OthmaneBen/dfe-task-t2extract-stacked-s43, OthmaneBen/dfe-task-t3summ-flat-s42, OthmaneBen/dfe-task-t3summ-stacked-s42, OthmaneBen/dfe-task-joint-s42, OthmaneBen/ qwen3.5-9b-t2-extract-stacked-wrongdomain-s42, OthmaneBen/qwen3.5-9b-wrongdomain-expert-r96, OthmaneBen/llama31-8b-t2-extract-flat-s42; Task datasets on Hugging Face: datasets/OthmaneBen/ dfe-stacking-datasets. An archived snapshot with DOI: 10.5281/zenodo.22649187. The domain corpus construction pipeline and license accounting are included in the code release; the built corpus is available from the authors for verification.

## Appendix A. Instrument Validation History

This appendix documents, in the order encountered, every evaluation-instrument defect found during the study, the evidence that localized it, and the fix. We include it because the paper’s central claim, that surface-form drift masquerades as capability change, was learned on our own instruments, and the record is itself evidence for the claim.

### Appendix A.1. Reasoning-Block Confound

The chat template opens a <think> block that the base model fills extensively (∼30% of generated tokens), while the fine-tuned expert, whose training data supervised an immediate close, does not. Under a fixed generation cap, this censors the two models asymmetrically: the base spends budget on reasoning, the expert on answers. The defect was detected via per-side token accounting and fixed by think-stripping in all graders plus a primed-close decoding protocol applied identically to both sides. Every subsequent evaluation uses this protocol.

### Appendix A.2. Grader Environment Gaps

The initial code-execution grader failed valid answers for environmental reasons: missing scientific stack libraries, SQL answers executed as Python, and blocking plt.show() calls. Harness fixes alone, without touching any model, moved the base model’s execution score from 29.8% to 55.3%, a measured bound on how much apparent model failure was instrument error. The final grader pre-loads the scientific stack, statically skips SQL only and unavailable framework items (symmetrically, by question), and neutralizes blocking calls.

### Appendix A.3. Stop-Token Misconfiguration

This is described in Section 4.8 and discovered here chronologically. Symptoms (ghost conversational turns, repetition, spurious truncation) initially resembled fine-tuning damage; a minimal-pair generation test isolated the upstream configuration gap. All subsequent generation passes the full stop list at the call site, and every saved artifact receives an authored generation_config.json.

### Appendix A.4. Merge Integrity (Ruled Out)

Because all graded evaluations use merged weights, we verified merge_and_unload fidelity by long greedy agreement between merged and unmerged assemblies: 8/10 exact continuations, with two late divergences consistent with BF16 associativity drift. Merging is not a confound.

### Appendix A.5. Register Localization

With the instrument repaired, the expert still trailed on keyword grading (67.8% vs. 88.6%). Three pre-registered checks localized the cause before any human re-grade: (i) no length gradient within the expert’s failures; (ii) missed keywords form a long tail of peripheral synonyms rather than content words; (iii) hand-read discordant items are factually correct paraphrases. This motivated the blind re-grade of Section 4.

### Appendix A.6. T1 Mining Leak (v1 → v2)

The v1 task-mining filter used an over-permissive disjunction (write.*function matched any language), admitting ∼22% non-Python items across all splits, detected when at-cap generations turned out to be Perl and C# answers to leaked questions. The v2 miner requires a Python ecosystem conjunct and an other-language negative filter; regex-detectable leakage fell from 66/300 to 0/300 test items. Both experts were retrained on v2; v1 results (base 15.2, flat 10.9, stacked 7.9 exec on the porous set, paired discordance again a coin flip) replicate v2’s qualitative structure and are retained as a robustness footnote.

### Appendix A.7. T3 Arithmetic Bound

Per-key decomposition of missed facts across all five conditions shows multi-operand derived quantities failing uniformly (top3_share_pct: 54/54 missed by *every* condition; error breakdown totals and shares: 98–100/100 by every condition): single forward pass arithmetic over many operands bounds all-facts ceilings identically across arms, which is a capability boundary, not a condition differentiator. The unseen table type additionally reveals a reporting convention gap in trained models (rarest_error omitted 58–67/100), which is the T3 analogue of T2’s inferential field finding; base and domain miss literal facts via paraphrase, with the register signature on yet another surface.

### Appendix A.8. The F2 Reclassification

The strict unseen-schema result was initially interpreted as generalization collapse and foundation buffering; per-item failure classification then showed 237/242 failures across conditions and seeds to be the single version prefix coercion, and the interpretation was revised to surface fidelity stabilization before publication. We document this deliberately: it is a worked instance of the paper’s thesis occurring on the paper’s own headline number, caught by the audit rule we now state as method—*every aggregate supporting a mechanism claim receives an automated per-item failure-mode classification plus a spot-read of each discovered mode*. One caveat from the same episode: naive containment checks mis-score non-numeric facts (Booleans, lists); the study’s own quick audit tooling initially did, so classifiers must respect each grader’s normalization.

## Appendix B. Reproducibility

The release is designed for exact reproduction of every number in this paper.

### Appendix B.1. Release Contents

(i) The three task datasets (T1 code v1 and v2, T2 extraction, T3 summarization) with SHA-256 manifests and, for T1, per-item source attribution as required by the source license; (ii) adapter weights for every condition and seed (15 adapters: the r=96 domain expert, the r=48 task and joint experts, the r=96 wrong-domain control expert, the task expert stacked on it, and the Llama 3.1 8B flat task expert); (iii) all training, mining/synthesis, evaluation, grading, table- and figure-generation code, including the perturbation-battery generator, the battery itself (240 items with convention annotations), and the generalized evaluator with per-item outputs for every evaluated condition, with a values.json snapshot allowing any number in this paper to be diffed against the artifacts; (iv) the environment specification with install order (the flash-linear-attention/transformers version interaction requires it). The domain corpus is not redistributed; its construction pipeline, per-source license accounting, and decontamination procedure are part of the released code, and the built corpus is available from the authors for verification. Per-item generation logs for the evaluation grid are regenerable from the released adapters and harnesses and are available from the authors on request.

### Appendix B.2. Reproducible Numerical Results

Every number and table in this paper is generated by a release script (gen_tables.py) from the per-item artifacts; a values.json snapshot allows diffing the paper against the artifacts at any time. No result is hand-transcribed.

### Appendix B.3. Inference Determinism

All evaluation is greedy; training seeds are recorded in per-run configuration files saved beside each checkpoint. The seed replication analysis (Section 4) is reproducible from the released adapters alone.

## Appendix C. Blind Re-Grade Protocol

The protocol controls identity and order bias while measuring the automated tier’s error in both directions.

### Appendix C.1. Queues

From the 500-item open-ended domain evaluation, three review queues were constructed: **Q1**, items the automated keyword tier failed for the expert but passed for the base (the expert’s putative regressions); **Q2**, the reverse discordants; **Q3**, a calibration sample of 120 concordant items (60 both-pass, 60 both-fail) to measure the automated tier’s error rate where it *agrees* with itself. Coverage of keyword-failures is near-census (156/161 expert, 52/57 base), so corrected estimates involve almost no extrapolation.

### Appendix C.2. Blinding

The review interface hides model identity, randomizes item order per session (recorded seed), and presents one answer at a time with the question and the automated tier’s keyword list withheld until after the verdict. Reviewer id, timestamp, and application version are logged per review.

### Appendix C.3. Verdicts and Flags

Verdict classes: correct, partially_correct, incorrect, unratable; confidence in three levels; free-text note; multi-select flags including outdated_but_correct.

### Appendix C.4. Temporality Rule

An answer is correct if it was accurate at any point within a five-year window (2021–2026) covering both the base model’s training data and the fine-tuning corpus vintage (Stack Exchange dump ≈mid-2022; documentation cloned August 2026); staleness is flagged, not penalized; genuinely unratable API history goes to unratable with a note. Observed outdated_but_correct rate: 0/273 reviews, where the rule was exercised in the opposite direction (one answer wrong even at training time was marked incorrect without the flag, correctly).

### Appendix C.5. Metrics

Artifact rate = share of reviewed automated failures rescued as correct or partially_correct, with Wilson 95% intervals; corrected keyword-tier estimates in two constructions (one-sided rescue only, conservative; two-sided additionally extrapolating Q3’s false-pass rate). Dashboard aggregates were independently recomputed from the raw review export and agree within rounding.

### Appendix C.6. Rater Validation

A second blind rater independently re-graded all 268 dual-rated items. Agreement: raw 3-class yielded 74.6%, with a Cohen’s κ [29] of 0.570; on the metric-relevant lenient binary (correct/partial vs. incorrect), the raw agreement was 91.8%, with a κ of 0.612; the quadratic-weighted κ was 0.654. The gap between high raw agreement and moderate κ is the documented prevalence paradox [30]: with both raters passing ≈88% of items, chance agreement is large and κ penalizes the skew; prevalence-adjusted statistics sit in the almost perfect band (PABAK 0.836; Gwet’s AC1 [31] 0.896). Disagreements concentrate on the partial credit boundary (49 of 68 correct ↔ partially-correct). The headline artifact rate independently replicates across raters (86.9% vs. 89.7% pre-adjudication). The 22 metric-relevant disagreements were resolved in a joint consensus session under a pre-committed boundary rubric with a conservative default-to-lower tie-break rule; zero tie-breaks were needed. All reported figures are item-level under consensus > rater-1 precedence; original reviews are immutable and released. The estimator construction, stated explicitly, is that artifact rates are computed over all reviewed keyword-failures regardless of queue; corrected one-sided estimates add reviewed rescues to automated passes, counting unreviewed keyword-failures as failures; two-sided estimates additionally multiply automated passes by the consensus Q3 pass-cell lenient rate (0.967, both sides).

## Appendix D. Extended Operational Characterization

This appendix records the operational findings not already detailed in Section 4.8; the stop-token misconfiguration, unmerged-generation cost, and merge-fidelity results are documented there in full and are not repeated here.

**Memory geometry.** Gradient checkpointing reduces peak training VRAM 4.5× (75.8 → 16.7 GB, NF4 r=32), far beyond typical transformer savings, attributable to DeltaNet’s recurrent state tensors. The r=96 BF16 domain expert trains in 46 GB at micro-batch 1 with paged 8-bit AdamW. Adapter parameters at r=16/48/96: 41.5 M/121.7 M/242.1 M.

**Serving engines.** At the time of the reported runs, vLLM rejects DoRA and fails KV-cache profiling on DeltaNet layers; SGLang serves the base but cannot load adapters against the nested configuration. Pure transformers+PEFT (used throughout) is the reproducible path.
